# Determination of Tumor Marker Screening for Lung Cancer Using ROC Curves

**DOI:** 10.1155/2024/4782618

**Published:** 2024-03-21

**Authors:** Xiaofeng Dou, Jiachen Lu, Yingying Yu, Yaohui Yi, Ling Zhou

**Affiliations:** School of Public Health, Dalian Medical University, Dalian 116044, Liaoning, China

## Abstract

**Introduction:**

Lung cancer ranks first among malignant tumors worldwide and is a leading cause of cancer-related mortality in both men and women. Combining tumor marker testing is a strategy to screen individuals at high risk of pulmonary cancer and minimize pulmonary cancer mortality. Therefore, tumor marker screening is crucial. In this study, we analyzed combinations of tumor markers for lung cancer screening using receiver operating characteristic (ROC) curve analysis.

**Methods:**

A retrospective descriptive study was conducted on patients diagnosed with lung cancer, as well as healthy and benign lung diseases, using data from the China Huludao Central Hospital database between January 2016 and July 2022. The *t*-test and ROC curve were utilized to assess the effectiveness of individual tumor marker and the combination of multiple tumor markers. Tumor markers are molecular products metabolized and secreted by tumor tissues, characterized by cells or body fluids. They serve as indicators of tumor stage and grading, monitor treatment response, and predict recurrence.

**Results:**

In this study, 267 healthy participants, 385 patients with benign lesions, and 296 patients with lung cancer underwent tumor marker screening. The sensitivity of five tumor markers—CEA, CYFRA21-1, NSE, pro-GRP, and CA125—was found to be <55%. This study revealed that a single tumor marker had limited value in lung cancer screening. However, combining two or more markers yielded varying area under the curves (AUC), with no significant impact on screening accuracy. The combination of CEA + CA125 demonstrated the highest accuracy for lung cancer screening in healthy participants. At a cutoff of 0.447 for CEA + CA125, the combination showed a sensitivity of 0.676 and specificity of 0.846 for lung cancer screening. Conversely, for patients with benign lung lesions, the optimal combination was CEA + NSE, with a cutoff of 0.393, yielding a sensitivity of 0.645 and specificity of 0.766 for lung cancer screening.

**Conclusion:**

The five tumor markers—CEA, CA125, CY211, NSE, GRP—show promising results in screening healthy individuals and patients with lung cancer. However, only CEA, NSE, and GRP effectively differentiate patients with benign lung lesions from those with lung cancer. A single tumor marker has limited utility in detecting and screening for lung cancer and should be combined with other tumor markers. CEA + CA125 emerges as a superior tumor marker for distinguishing healthy individuals from those with lung cancer, whereas the CEA + NSE combination is more effective in identifying tumor markers in patients with benign lung lesions and lung cancer.

## 1. Background

Lung cancer ranks as among the most prevalent malignant tumors worldwide, with an annual incidence of approximately 1.82 million cases and 386,000 deaths, predominantly affecting men. The causes of lung cancer are multifactorial, including environmental factors such as air pollution, smoking, genetic predisposition, chronic lung diseases, and occupational exposures [1]. Due to its high incidence and poor prognosis, pulmonary carcinoma is a significant contributor to cancer-related mortality globally, posing a growing disease burden in many countries [2]. In China alone, lung cancer accounts for about 787,000 annually [3], making it the most prevalent cancer and a leading cause of cancer-related deaths, with a 12.16% increase in incidence over the past decade [4]. Efforts to address the escalating burden of cancer in China face challenges due to the rapid aging of the population and cumulative exposure to risk factors [5]. If detected at an early stage, surgical resection of lung cancer offers a favorable prognosis, with a 5-year survival rate of 70%–90% for small localized tumors. However, most patients (approximately 75%) are diagnosed with advanced disease, and despite recent advancements in oncological treatment, survival rates remain low [6]. Early detection through screening is crucial for improving the survival rate of patients with lung cancer. Although various screening methods exist, early detection remains challenging [7].

Therefore, the timely identification of pulmonary cancer is imperative. Screening tests for lung cancer play a crucial role in identifying patients at an early stage, facilitating potentially curative local resection. Tumor markers present in clinical specimens hold promise for reducing lung cancer mortality. Furthermore, in cases where cancer is diagnosed at an advanced stage, sensitive molecular markers may can aid in clarifying the diagnosis without necessitating additional invasive diagnostic procedures. Early diagnosis and confirmation of tumor biomarkers can improve the outcomes of lung cancer treatment. The precise early detection of tumor biomarkers can enhance the effectiveness of pulmonary cancer diagnosis and lung cancer detection. Simultaneously, early screening can reduce the potential damage to individuals who are less likely to develop lung cancer [8]. The measurement or identification of tumor markers, which are molecules that occur in the blood and tissues and are related to cancer, or are the result of a host response to cancer, is highly valuable for clinical diagnosis and patient management [9]. Neurocyte-specific enolase (NSE), carcinoembryonic antigen (CEA), cytokeratin fragment (CYFRA21-1), pro-gastrin-releasing peptide (pro-GRP), and carbohydrate antigen 125 (CA125) are routine markers recommended by the National Academy of Clinical Biochemistry (NACB) guidelines for laboratory medicine in lung cancer. However, these five indicators were not systematically assessed in the same cohort [10].

CEA is a serum glycoprotein commonly used as a marker for lung, breast, and colorectal cancers. Normally absent in healthy adults, CEA exists as a ligand for L-selectin and E-selectin. Increased CEA concentrations are observed in smokers and individuals with nonneoplastic illnesses [11]. CA125 is a mucus glycoprotein primarily associated with ovarian cancer [12], but it is also utilized in monitoring cancer progression and has emerged as a primary biomarker screening candidate [13]. Serum CA 125 concentration correlates with TNM stage, histological type, and survival rate in patients with lung cancer [14]. CYFRA21-1, a cytokeratin 19 soluble fragment, is a newly discovered monoclonal antibody with comparative diagnostic value in detecting nonsmall cell lung cancer (NSCLC), particularly in patients with squamous cell carcinoma (SCC) [15]. Serum CYFRA21-1 shows promise as a biomarker for differentiating small cell lung cancer (SCLC) from NSCLC [16]. NSE, also known as enolase 2 (ENO2), is a member of the enolase family expressed by mammalian neurons and neuroendocrine cells [17]. NSE is already utilized as a biomarker in the differential diagnosis of SCLC. Additionally, NSE holds particular prognostic value for predicting outcomes in patients with severe craniocerebral trauma [18]. NSE is a well-established marker, and its serum levels are utilized to support the initial diagnosis of SCLC. Numerous studies have demonstrated that NSE exhibits high diagnostic power in patients with SCLC [19]. Gastrin-releasing peptide (GRP) was first isolated from the stomach of pigs in 1978 and has since been found to be distributed in various organs and tissues, including the fetal lung and lung cancer, with good sensitivity and specificity [20]. In healthy adult participants, GRP levels increase with age, and smokers typically exhibit higher concentrations compared with nonsmokers. Additionally, GRP levels rise rapidly in patients with SCLC during advanced stages of the disease [21]. Biomarkers of lung pose challenges in terms of sensitivity and specificity, hindering their effectiveness in detection and diagnosis [22]. Due to the sensitivity and specificity of individuals biomarkers, various biomarker methods have been developed for cancer detection instead of relying solely on a single biomarker approach [23].

Most studies have primarily focused the test results of individual tumor markers in lung cancer screening, with limited investigation into the combined detection of tumor markers [24]. Conversely, this study examined the efficacy of combined detection using various tumor markers, which is critical for selecting optimal markers for lung cancer screening [25]. By using receiver operating characteristic (ROC) curves, the cutoff values for marker combinations can be adjusted to achieve a balance between sensitivity and specificity. However, in other studies, fixed cutoff values were utilized, resulting in sensitivity and specificity levels that may not always meet the requirements for effective screening [26].

Nevertheless, screening for various tumor markers may pose potential risks, including a higher rate of false positives, decreased specificity, and other hazards [27]. Therefore, although cotesting with tumor markers has been acknowledged as an effective diagnostic aid for tumors, it cannot be assumed that utilizing more markers leads to greater diagnostic efficiency [28]. To date, no organization has thoroughly examined which tumor markers should be used in conjunction with screening, both in healthy individuals and those with benign lung disease. In this study, we utilized a combination of ROC curves and binary logistic regression analysis to identify the optimal combination of markers suitable for detecting pulmonary carcinoma.

## 2. Materials and Methods

### 2.1. Patients

This case–control study was conducted at Huludao Central Hospital, Huludao city, Liaoning province, between January 2016 and July 2022. The study included all patients who underwent physical examinations for lung cancer at the hospital during the 6-year period. We also included 296 patients with lung cancer, comprising 149 males (50.33%) and 147 females (49.66%), with a mean age of 62.93 ± 11.12 years. Additionally, 384 patients with benign pulmonary lesions were included, with a mean patient age of 67.09 ± 14.28 years, including 188 males (48.9%) and 196 females (51.0%). Benign lung lesions encompassed conditions such as pneumonia, lung infection, chronic obstructive pulmonary disease, and bronchial asthma. Pulmonary samples were obtained from 267 healthy controls, of which 131 were male (49.0%) and 136 were female (51.0%), with a mean age of 58.82 ± 12.79 years. All selected participants provided informed consent, and the study received approval from the Institutional Review Board of the hospital.

### 2.2. Methods

This retrospective descriptive research utilized available clinical data to summarize and analyze retrospectively [29]. The advantages of this approach include requiring fewer human resources, minimal funding requirements, noninterference with normal clinical work, and a short research period. In this retrospective cross-sectional study, data were collected from individuals who had undergone lung cancer screening or treatment at our hospital over the past 6 years, including 267 healthy individuals, 384 patients with benign lung lesions, and 296 patients with lung cancer. These data were collated and analyzed retrospectively, and the selection of tumor markers for lung cancer screening was analyzed using ROC curves and binary logistic regression to identify for the most effective tumor marker combinations. All participants were administered 4 mL of fasting venous blood, centrifuged at 3,000 r/min for 10 min. The levels of CEA, CYFRA21-1, SCC-Ag, pro-GRP, and CA125 were measured using a chemiluminescence instrument, strictly following the manufacturer's instructions. The following reference ranges were applied: CEA > 5 ng/mL, CA125 > 35 U/mL, CYFRA21-1 > 3.3 ng/mL, pro-GRP > 65.7 pg/ml, and NSE > 16.3 ng/mL.

SPSS.21 software (IBM Corp. Released 2012. IBM SPSS Statistics for Windows, Version 21.0. Armonk, NY: IBM Corp.) and MedCalc® Statistical Software version 19.6.4 (MedCalc Software Ltd., Ostend, Belgium; https://www.medcalc.org; 2021) were employed for statistical analysis of the data in this study. Continuous variables were expressed as mean ± standard deviation (*x* ± *s*), and comparisons of changes in tumor marker concentrations between groups were performed using *t*-test or *chi*-square analysis. Significant differences were considered when *P* <0.05. The predictive probability of lung cancer diagnosis by combining tumor markers was assessed using binary logistic regression, and ROC curves were generated with the predictive probabilities as variables. The area under curve (AUC) was calculated as the area enclosed by the coordinate axis under the ROC curve and was utilized to evaluate the statistically significance of each marker [30]. The ROC curve is a graphical representation of true positive rate versus false positive rate (FPR) for various threshold settings. The ROC curve is a function of sensitivity and FPR, playing a central role in assessing the diagnostic power of a test to differentiate between the true state of a participant, determine the optimal cutoff value, and compare two alternative diagnostic tasks when each task is performed on the same participant [31]. ROC curves are extensively employed in clinical epidemiology to assess the diagnostic efficacy of serum markers and differentiate between diseased and healthy participants. ROC curves were employed to assess the diagnostic efficacy of serum markers and classify the tests between diseased and healthy participants, a method widely used in clinical epidemiology. When the AUC is >0.7 it indicates that the marker has good diagnostic value. An AUC ranging from 0.5 to 0.7 indicated an average diagnosis. However, when the area was <0.5, the diagnosis was not statistically significant [32].

## 3. Results

**Figure 1:**
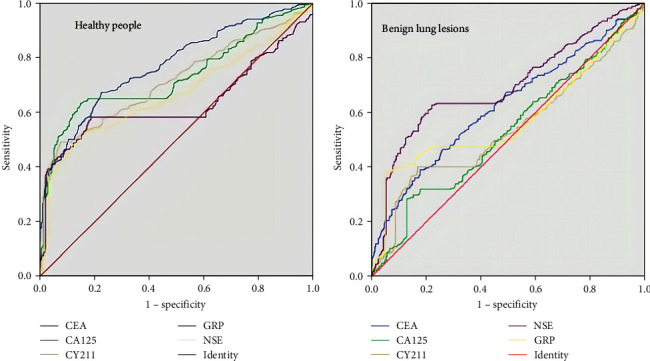
ROC curves for single tumor markers in different populations: (a) healthy participant and (b) benign lung lesions.

Implications of single tumor markers for lung cancer screening. A *t*-test analysis of the data from all tests in this study (Table 1) revealed that the concentrations of all five tumor markers, including CEA (*P* <0.001), CA125 (*P*=0.003), NSE (*P*=0.023), GRP (*P* <0.001), and CY211 (*P* <0.001), were dramatically different when lung cancer tumor marker testing was performed in healthy populations versus lung cancer patients. Conversely, when comparing participants with benign lung disease with those with pulmonary carcinoma, no statistically significant variation was noted in the concentrations of the two tumor markers, CA125 (*P*=0.46) and CY211 (*P*=0.23). In contrast, the concentrations of the tumor markers CEA (*P* <0.001), NSE (*P* <0.001), and GRP (*P* <0.001) were significantly higher in patients with pulmonary cancer than in those with benign lung disease. This study also compared the ages of healthy individuals, patients with benign lung lesions, and patients with lung cancer and found that healthy individuals tended to be younger than patients with lung cancer (*P* <0.001), whereas patients with benign lung lesions tended to be older than patients with lung cancer (P <0.001). As shown in Figure 1, ROC curves were constructed with the five tumor markers as test variables for the healthy group and patients with benign lung cancer, and the AUC was calculated. The diagnostic efficacies of various tumor markers calculated using the ROC curve are displayed in Table 2. In a comparison between the healthy population and patients with lung cancer, CEA, CA125, and CY211 had good diagnostic effects in lung cancer screening (AUC > 0.7), whereas NSE and GRP had limited diagnostic value for pulmonary cancer (AUC < 0.7). For patients with benign lung lesions, CA125 and CY211 had no application in lung cancer screening (AUC < 0.5), and the diagnostic importance of the remaining three markers remained weak (AUC < 0.7).

**Figure 2:**
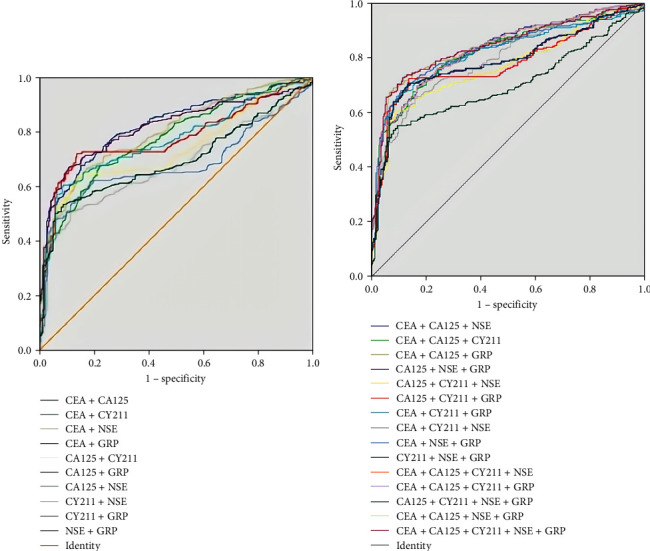
ROC curves for combined tumor marker screening in healthy populations: (a) two tumor markers and (b) multiple tumor markers.

Comparison of the sensitivity, specificity, Jorden index, and AUC of combined tumor markers in lung cancer screening in a healthy population. ROC curves were established by analyzing tumor marker pulmonary cancer screening in healthy individuals using multivariate binary logistic regression. Table 3 illustrates the lung cancer screening performed using the biomarkers in healthy individuals, whereas the corresponding ROC curves are displayed in Figure 2. The results revealed that 10 permutations (CEA + CA125, CEA + CA125 + NSE, CEA + CA125 + CY211, CEA + CA125 + GRP, CEA + NSE + GRP, CEA + CA125 + CY211 + NSE, CEA + CA125 + CF21 + GRP, CA125 + CY211 + NSE + GRP, CEA + CA125 + NSE + GRP, and CEA + CA125 + CFA21 + NSE + GRP) exhibited significantly higher AUCs than the other combinations, with negligible differences observed among their AUCs. No obvious discrepancy was found between the minimal AUC (CEA + CA125; marked with ▴) and the maximal AUC (CEA + CA125 + CY 211 + NSE + GRP; marked with ★) among these 10 combinations (*Z* = 1.684, *P*=0.09). The sensitivity (*P* <0.001) and negative predictive value (*P* <0.001) of CEA + CA125 were lower than those of CEA + CA125 + CY211 + NSE + GRP, whereas the specificity (*P* <0.001) and positive predictive value (*P* <0.001) were significantly higher. The Jorden index for CEA + CA125 was also lower than that for CEA + CA125 + CY211 + NSE + GRP.

**Figure 3:**
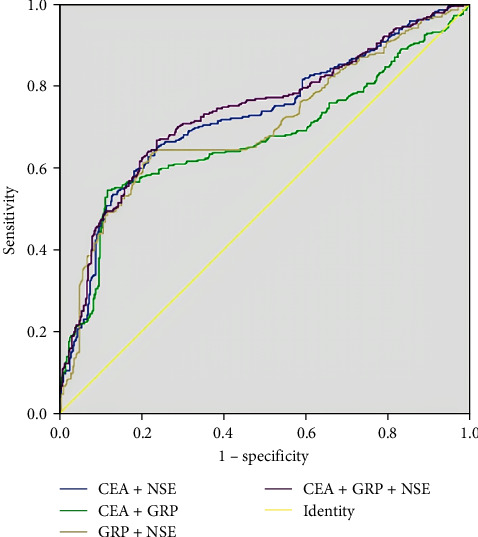
ROC curves for tumor markers in benign lung disease.

The ROC curves of the combined pulmonary carcinoma screening markers in patients with benign lung disease are depicted in Figure 3. The combinations of multiple markers employed for the evaluation of lung cancer detection are shown in Table 4. Because CA125 and CY211 did not contribute significantly to differentiating benign lung lesions from lung cancer, these two tumor markers were not included in our analysis. As the results indicated, two combinations (CEA + NSE and CEA + NSE + GRP) exhibited an AUC > 0.7, and no obvious difference was noted between the comparison of AUC of CEA + NSE (marked with ▴) and CEA + NSE + GRP (marked with ★; *Z* = 1.019, *P*=0.308). The sensitivity of CEA + NSE was lower than that of CEA + NSE + GRP (*P* < 0.001), but its specificity was higher than that of CEA + NSE + GRP (*P* <0.001). Moreover, the differences in the PPV and NPV were not significant (*P*=0 − 610 and 0.163), with the latter having a higher Jorden index than the former.

The study examined the impact of age, sex, and various other factors on the selection of tumor markers for lung cancer. According to the World Health Organization (WHO), individuals aged >65 years are considered older adults [33]. In this investigation, participants were categorized based on their age, and the cutoff value was 65 years. Each group was further subdivided into by sex. Finally, patients with pulmonary cancer or benign lung disease and healthy participants were classified into 12 panels. Individual subgroup sizes are displayed in Table 5. To investigate the effects of seven factors, including NSE, GRP, CA125, CY211, CEA, age, and sex, on lung cancer screening status, we performed a multivariate binary logistic regression analysis with these seven factors as independent variables and lung cancer screening status as the dependent variable. For this study, age = 1 for individuals aged <65 years and age = 2 for those aged ≥65 years.

The logistic regression model included sex, age, CEA, CYFRA21-1, NSE, pro-GRP, and CA125 as independent variables, with the presence or absence of the disease as the dependent variable coded as 0 for no and 1 for yes. Binary logistic regression revealed that in healthy participants, CEA (*P* <0.001), CA125 (*P* <0.001), GRP (*P*=0.001), and age (*P*=0.004) were significant factors affecting lung cancer screening. However, sex (*P*=0.141), CY211 (*P*=0.827), and NSE (*P*=0.078) levels did not significantly influence lung cancer screening results in healthy participants. Conversely, for patients with benign lung lesions, CEA (*P* <0.001), NSE (*P*=0.001), GRP (*P*=0.013), and age (*P* <0.001) were influential factors in lung cancer screening, whereas sex (*P*=0.81), CA125 (*P*=0.22), and CY211 (*P*=0.33) did not significantly impact the screening of patients with benign lung lesions. Further details are presented in Tables 6 and 7.

**Figure 4:**
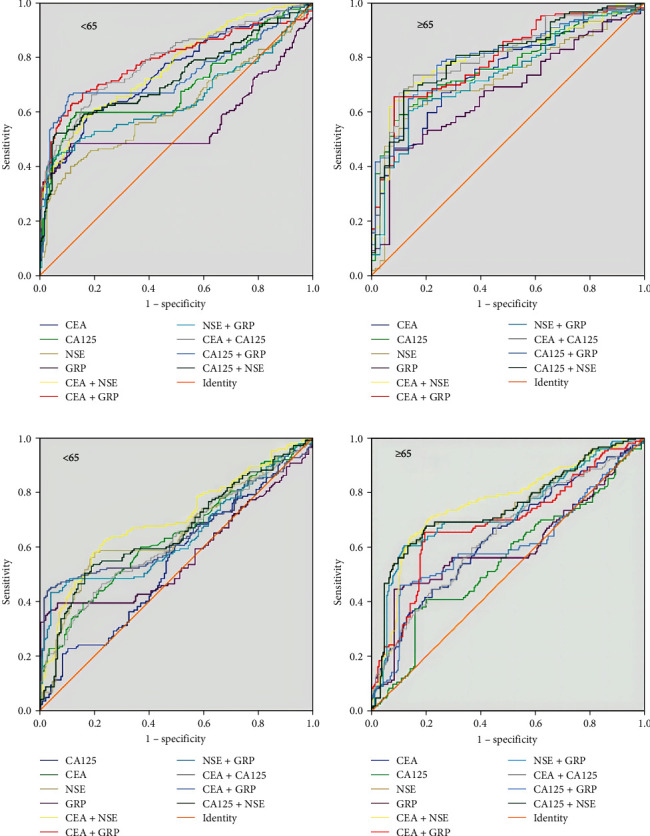
ROC curves for tumor markers in lung cancer screening across different age groups: (a) healthy individuals aged <65 years, (b) healthy individuals aged ≥65 years, (c) patients with benign lung lesions aged <65 years, and (d) patients with benign lung lesions aged ≥65 years.

To analyze the screening of tumor markers for lung cancer diagnosis, we divided healthy participants and patients with benign lung lesions into age-based cohorts and analyzed their ROC curves. The AUCs of various combinations of tumor markers for lung cancer diagnosis in different populations are shown in Table 8. The calculated ROC curve is presented in Figure 4. We calculated the AUC values based on each group, identified the top three combinations (marked in bold), and compared their AUC values to determine the optimal tumor marker combination for lung cancer screening in different groups (a1, a2, a3, d1, d2, and d3). In healthy participants, the CEA + CA125 combination displayed the largest AUC value among all combinations, regardless of age, and CEA + CA125 is evidently the optimal tumor marker combination for lung cancer screening among healthy participants. Conversely, CEA + NSE is the optimal combination for screening for lung cancer in patients with benign lung diseases. Age is crucial for both the differentiation of healthy individuals from those with lung cancer, and the differentiation of patients with benign lung disease from those with lung cancer.

The cutoff values for the optimal marker combinations for lung cancer screening and their corresponding sensitivity and specificity, along with the results of pulmonary cancer screening utilizing the combinations of CEA + CA125 and CEA + NSE in healthy participants and patients with benign lung diseases are outlined in Table 9. The sensitivity of each combination (SEN) and its corresponding specificity (SPE) were measured using the maximum Jorden index. In healthy individuals, CEA + CA125 displayed a sensitivity of 0.676 and specificity of 0.846 for lung cancer at a cutoff value of 0.447. Conversely, for patients with benign lung disease, CEA + NSE demonstrated a sensitivity of 0.645 and a specificity of 0.766 for lung cancer at a cutoff value of 0.393.

Comparison with other lung cancer tumor marker selection studies reveals that this study employed multivariate binary logistic regression analysis and ROC curves to select tumor marker combinations for lung cancer screening, a method informed by previous research. Unlike other studies where threshold values for tumor markers were derived from reagent company recommendations without ROC curve analysis, this study rigorously determined cutoff values using ROC curves. This study identified CEA + CA125 as the most effective combination for lung cancer screening in healthy individuals, with a sensitivity of 0.676 and specificity of 0.846. Compared with studies without ROC curve analysis, this approach resulted in significantly higher sensitivity (*P*=0.001), whereas specificity remained relatively unchanged (*P*=0.133). Similarly, for patients with benign lung lesions, CEA + NSE emerged as the optimal tumor marker combination, with a sensitivity of 0.6453 and specificity of 0.7662. Compared with nonROC curve methods, this approach led to significantly higher sensitivity (*P*=0.025) but lower specificity (*P* <0.001). All findings are shown in Table 10.

## 4. Discussion

Lung cancer stands as the most common malignant tumor in China and carries the highest mortality rate among all malignant tumors [34]. Therefore, conducting lung cancer screening is crucial. To the best of our knowledge, this is the first screening study to explore tumor marker binding between healthy patients and those with pulmonary cancer, as well as between patients with benign lung lesions and those with lung cancer.

Although the selection of tumor markers for lung cancer has remained stable over the past 10 years, some changes have occurred. The 2011 guidelines recommended the use of CEA, NSE, CYFRA21-1, and SCC [35]. The primary lung cancer markers recommended in the 2015 lung cancer guidelines include CEA, NSE, CYFRA21-1, pro-GRP, and SCC [36]. In the 2018 lung cancer guidelines, the recommended tumor markers did not change [37].

In this study, we aimed to assess the efficacy of tumor markers in detecting lung cancer. CEA, CY211, CA125, NSE, and GRP are relevant tumor markers for lung cancer [38]. These markers are automated and suitable for large-scale lung cancer detection [39]. To identify the optimal combination for pulmonary cancer screening, ROC curves were constructed, and the diagnostic value was evaluated using the AUC. Logistic regression was employed to consolidate various markers into a single variable for ROC curve analysis, and ROC curves were subsequently generated based on these probabilities.

Five tumor markers, namely CEA, CY211, CA125, NSE, and GRP, exhibited significance in distinguishing healthy patients from those with lung cancer. Conversely, CEA, NSE, and GRP demonstrated significance in discriminating benign lung lesions from lung cancer, whereas CA125 and CY211 did not. CEA, CA125, and CY211 were effective in differentiating healthy participants from those with lung cancer (AUC > 0.7), whereas NSE and GRP were not (AUC < 0.7). However, none of the tumor markers were effective in differentiating benign lung disease from lung cancer (AUC < 0.7). These findings indicate that the detection of a single tumor marker alone is insufficient for the diagnosis of lung cancer.

Therefore, this study utilized combinations of tumor markers for lung cancer screening. To explore the diagnostic efficacy of each combination, the results were analyzed using binary logistic regression and ROC curve analyses. In the healthy population, the AUC increased with an increase in tumor markers. However, this does not imply that more tumor markers in the mix are necessarily better. Sensitivity and specificity are known to be inversely related, as sensitivity increases, specificity tends to decrease. Among the 10 combinations selected with an AUC > 0.820, only the CEA + CA125 combination comprised two markers, where the remaining combinations consisted of various markers. Typically, when no significant difference exists in the diagnostic value between a larger number of combinations and a smaller number of combinations for lung cancer screening, a smaller number of combinations should be selected for testing [40]. This study compared the AUC values between different tumor markers in a healthy population using MedCalc software and found no significant differences between CEA + CA125 and other combinations, aligning with previous research findings. Furthermore, the sensitivity and PPV of CEA + CA125 were found to be the highest among all combinations in this study. Consequently, we concluded that dual tumor markers are superior to multiple tumor markers for lung cancer screening in healthy populations. CEA + CA125, in particular, emerged as the most effective of combination among all tumor marker combinations and should be prioritized for use. We applied a similar approach for lung cancer screening in patients with benign lung lesions. Our analysis revealed only two combinations with an AUC > 0.7: CEA + NSE and CEA + NSE + GRP. Notably, no significant difference was observed in the AUC between these two combinations, whereas the specificity of CEA + NSE was higher than that of CEA + NSE + GRP. Therefore, we concluded that CEA + NSE represents the optimal combination for the selection of tumor markers for lung cancer screening in patients with benign lung lesions. Other studies have corroborated the effectiveness of the CEA + CA125 combination in screening for lung cancer among healthy individuals. To the best of our knowledge, this is the first study to examine the combination of tumor markers for screening lung cancer in patients with benign lung disease.

Next, we investigated the tumor marker combinations using multivariate binary logistic regression and ROC curves. We analyzed the diagnostic efficacy of the markers across different age and sex groups. Our investigation revealed studies that used binary logistic regression to demonstrate the value of CEA, CA125, pro-GRP, and age in distinguishing between healthy patients and those with lung cancer. In contrast, CEA, NSE, pro-GRP, and age were deemed valuable in the diagnosis of patients with benign pulmonary lesions. Given our finding that dual tumor markers outperformed multiple tumor markers in lung cancer screening, we focused our ROC curve analysis solely on individual and paired markers. Notably, regardless of sex and age, CEA + CA125 emerged as the optimal choice for screening healthy individuals. When the cutoff point of CEA + CA125 was set at 0.447, this combination exhibited sensitivities of 0.676 and 0.846, respectively. In patients with benign lung lesions, age and sex did not affect the combination choice, with CEA + NSE proving to be the most efficacious option. With a cutoff value of 0.393 for CEA + NSE, the combination displayed a sensitivity of 0.645 and specificity of 0.766 for lung carcinoma screening. Importantly, age and sex had no impact on the selection of tumor marker combinations, whether in screening healthy individuals and patients with lung cancer or in screening benign lung lesions versus lung cancer. Compared with other studies that did not use ROC curve analysis, our study demonstrated a remarkable enhancement in sensitivity while maintaining a relatively stable specificity when screening for pulmonary carcinoma in healthy individuals. However, in patients with benign lung lesions, the sensitivity was significantly higher, whereas the specificity was significantly lower. One significant advantage of our study is that the ROC curve can be employed to adjust the cutoff values of tumor markers to balance sensitivity and specificity. In contrast, previous studies often used fixed cutoff values, making it challenging to meet the required sensitivity and specificity criteria.

The focus of our study differs from that of other lung cancer screening studies. In other lung cancer screenings, the discussion focused on the sensitivity and specificity of the marker combinations without specifying the tumor markers selected for combination and lung cancer screening. Consequently, researchers may experience uncertainty when selecting tumor marker combinations. This study seeks to address this gap by estimating the diagnostic value through the comparison of correlations between different combinations of tumor markers and clinical symptoms.

This study employed a cross-sectional approach, which has inherent limitations in establishing causal relationships. Future research should prioritize longitudinal exploration. Longitudinal research methods can help estimate the longitudinal trajectory characteristics of changes in individuals over time [41]. By extracting individual-specific longitudinal features, longitudinal prediction models containing more information have been proposed. These models enable clinicians to make more accurate predictions by leveraging repeated measurements of tumor markers. A longitudinal model can facilitate dynamic predictions by considering the time dependance of extracted features. For targeted patients, the predicted risk can be continuously updated over time to reflect the potential changes in prognosis.

In this study, five serological markers were used to identify the optimal combination for lung cancer screening across various groups. The study also assessed the optimal combination cutoff values, sensitivity, and specificity through a combination of ROC curves and logistic regression analysis. Our findings indicate that using dual markers outperforms the use of multiple markers for serological tumor marker screening. The combination of ROC curves and logistic regression analysis proves to be effective in identifying neoplastic markers. Furthermore, this study suggests that the method developed herein may have promising applications for tumors beyond lung cancer and other non-neoplastic diseases.

## 5. Conclusion

The five tumor markers, CEA, CA125, CY211, NSE, and GRP, demonstrated validity in screening both healthy individuals and patients with pulmonary carcinoma. However, only CEA, NSE, and GRP levels were effective in distinguishing patients with benign lung lesions from those with lung cancer. Conversely, single tumor markers showed limited effectiveness in lung cancer screening, highlighting the necessity of combined tumor marker testing. Specifically, CEA + CA 125 proved effective in identifying lung cancer in healthy patients, whereas CEA + NSE was effective in identifying benign lung lesions in patients with lung cancer.

## Figures and Tables

**Table 1:** Serum marker concentrations and age in patients with lung cancer and benign lung lesions and in healthy subjects.

	Healthy	Lung cancer	*P*
Year	54.82 ± 12.79	62.93 ± 11.12	<0.001
CEA (ng/mL)	5.87 ± 3.20	37.43 ± 128.73	<0.001
CA125 (U/mL)	13.95 ± 16.56	56.06 ± 138.34	0.003
CY211 (ng/mL)	13.95 ± 16.56	8.57 ± 31.12	0.023
GRP (pg/ml)	48.09 ± 27.11	95.52 ± 158.82	<0.001
NSE (ng/mL)	14.15 ± 7.10	20.75 ± 30.19	<0.001

	Benign lung disease	Lung cancer	*P*

Year	67.09 ± 14.28	62.93 ± 11.12	<0.001
CEA (ng/mL)	5.85 ± 16.31	37.43 ± 128.73	<0.001
CA125 (U/mL)	44.09 ± 106.99	56.06 ± 138.34	0.46
CY211 (ng/mL)	5.96 ± 26.21	8.57 ± 31.12	0.23
GRP (pg/ml)	57.88 ± 52.42	95.52 ± 158.82	<0.001
NSE (ng/mL)	14.14 ± 10.74	20.75 ± 30.19	<0.001

**Table 2:** The value of single tumor marker testing in screening for lung cancer in patients with benign lung lesions and healthy individuals.

	SEN	SPE	PPV	NPV	+LR	−LR	Yden	Cutoff	AUC
Benign lung disease									
CEA	0.501	0.690	0.555	0.642	1.61	0.72	0.212	5.625	0.619
CA125	0.346	0.672	0.449	0.571	1.03	0.97	0.154	54.56	0.537
NSE	0.481	0.877	0.752	0.686	3.91	0.59	0.405	13.94	0.695
GRP	0.471	0.805	0.651	0.663	2.41	0.65	0.329	90.01	0.570
CY211	0.511	0.519	0.451	0.579	1.06	0.94	0.232	4.49	0.538
Healthy									
CEA	0.500	0.890	0.836	0.616	4.54	0.56	0.448	2.335	0.771
CA125	0.340	0.970	0.927	0.573	11.33	0.68	0.483	14.61	0.729
NSE	0.483	0.880	0.812	0.613	4.02	0.58	0.362	16.42	0.667
GRP	0.469	0.936	0.891	0.614	7.32	0.56	0.395	48.55	0.663
CY211	0.510	0.857	0.798	0.612	3.56	0.57	0.417	3.62	0.713

SEN: sensitivity; SPE: specificity; PPV: positive predictive value; NPV: negative predictive value; +LR: positive likelihood ratio; −LR: negative likelihood ratio; and Yden: Youden index.

**Table 3:** Lung cancer screening is performed with the biomarkers in healthy people.

Healthy	SEN	SPE	PPV	NPV	+LR	−LR	Yden	AUC
CEA + CA125^▴^	0.601	0.865	0.832	0.661	4.44	0.46	0.523	0.821
CEA + CY211	0.676	0.764	0.761	0.680	2.86	0.424	0.455	0.776
CEA + NSE	0.693	0.794	0.789	0.699	3.36	0.386	0.494	0.798
CEA + GRP	0.730	0.850	0.844	0.739	4.86	0.205	0.561	0.814
CA125 + CY211	0.595	0.835	0.800	0.650	3.57	0.277	0.489	0.733
CA125 + GRP	0.619	0.838	0.888	0.683	3.82	0.261	0.582	0.774
CA125 + NSE	0.585	0.850	0.813	0.648	3.90	0.488	0.516	0.764
CY211 + NSE	0.67	0.752	0.750	0.672	2.68	0.438	0.388	0.678
CY211 + GRP	0.713	0.812	0.809	0.718	3.79	0.353	0.422	0.677
NSE + GRP	0.663	0.831	0.814	0.689	3.92	0.405	0.445	0.694
CEA + CA125 + NSE	0.723	0.767	0.776	0.714	3.10	0.361	0.528	0.829
CEA + CA125 + CY211	0.710	0.741	0.753.	0.697	2.74	0.391	0.523	0.821
CEA + CA125 + GRP	0.760	0.827	0.830	0.756	4.39	0.290	0.607	0.838
CA125 + NSE + GRP	0.723	0.808	0.807	0.724	3.76	0.257	0.562	0.785
CA125 + CY211 + NSE	0.717	0.730	0.747	0.689	2.65	0.387	0.512	0.764
CA125 + CY211 + GRP	0.754	0.794	0.802	0.743	2.79	0.309	0.585	0.776
CEA + CY211 + GRP	0.804	0.730	0.768	0.770	2.97	0.335	0.561	0.816
CEA + CY211 + NSE	0.771	0.674	0.724	0.725	2.36	0.339	0.492	0.797
CEA + NSE + GRP	0.804	0.760	0.788	0.777	3.35	0.257	0.571	0.824
CYF21+NSE + GRP	0.787	0.715	0.754	0.751	2.74	0.297	0.454	0.698
CEA + CA125 + CY211 + NSE	0.787	0.651	0.715	0.734	2.04	0.327	0.528	0.828
CEA + CA125 + CY211 + GRP	0.814	0.711	0.758	0.775	2.86	0.260	0.611	0.838
CA125 + CY211 + NSE + GRP	0.855	0.647	0.751	0.800	2.40	0.224	0.571	0.824
CEA + CY211 + NSE + GRP	0.855	0.647	0.729	0.800	2.42	0.219	0.568	0.785
CEA + CA125 + NSE + GRP	0.814	0.737	0.775	0.781	3.09	0.252	0.614	0.840
CEA + CA125 + CY211 + NSE + GRP^★^	0.858	0.629	0.720	0.800	2.31	0.225	0.611	0.840

**Table 4:** The value of various combinations of lung cancer screening markers in patients with benign lung lesions.

Benign lung disease	SEN	SPE	PPV	NPV	+LR	−LR	Yden	AUC
CEA + NSE^▴^	0.693	0.620	0.585	0.724	1.829	0.494	0.410	0.723
CEA + GRP	0.730	0.584	0.575	0.737	1.758	0.461	0.426	0.667
GRP + NSE	0.663	0.719	0.645	0.734	2.365	0.468	0.407	0.697
CEA + GRP + NSE^★^	0.804	0.525	0.563	0.781	1.719	0.367	0.427	0.734

**Table 5:** Number of people grouped according to gender and age.

	Lung cancer	Benign lung disease	Healthy
Male			
≥65	73	126	33
<65	76	62	98
Female			
≥65	66	97	25
≤65	81	99	111
Total	296	384	267

**Table 6:** Binary logistic regression for lung cancer screening in healthy participants.

Variables	*B*	*P*	OR	95% C.I.
Gender	−0.305	0.141	0.73	0.492–1.106
CEA	0.0180	<0.001	1.19	1.098–1.306
CA125	0.026	<0.001	1.02	1.013–1.041
CY211	−0.001	0.827	0.99	0.990–1.008
NSE	0.027	0.078	1.02	0.997–1.059
GRP	0.013	0.001	1.01	1.006–1.021
Age	−0.648	0.004	0.52	0.338–0.810

**Table 7:** Binary logistic regression for lung cancer screening in patients with benign lung lesions.

Variables		*B*	*P*	OR	95% C.I.
a	Gender	0.039	0.81	1.04	0.750–1.443
	CEA	0.019	<0.001	1.01	1.010–1.029
	CA125	−0.001	0.22	0.99	0.971–.001
	CY211	−0.004	−0.33	0.99	0.989–1.004
	GRP	0.004	0.013	1.00	1.001–1.008
	NSE	0.039	0.001	1.04	1.015–1.066
	Age	0.588	<0.001	1.80	1.294–2.506

**Table 8:** AUC for various combinations of markers to screen for lung cancer in different populations at different ages.

	Healthy		Benign lung disease	
Year	<65	≥65	<65	≥65
CEA	0.746	0.742	0.632	0.640
NSE	0.615	0.726	**0.652** ^**c1**^	**0.743** ^**d1**^
GRP	0.562	0.672	0.568	0.601
CA125	0.694	0.768	0.542	0.549
CEA + NSE	**0.762** ^**a1**^	**0.800** ^**b1**^	**0.697** ^**c2**^	**0.759** ^**d2**^
CEA + GRP	**0.785** ^**a2**^	0.794	0.647	0.685
NSE + GRP	0.644	0.733	0.637	0.729
CEA + CA125	**0.785** ^**a3**^	**0.808** ^**b2**^	0.625	0.644
CA125 + GRP	0.735	0.798	0.585	0.603
CA125 + NSE	0.721	**0.800** ^**b3**^	**0.652** ^**c3**^	**0.743** ^**d3**^

a1 vs a2: *Z* = 1.202 *P*=0.2293; a1 vs a3: *Z* = 1.445 *P*=0.1484 ; a2 vs a3: *Z* = 0.004 *P*=0.9964; b1 vs b2: *Z* = 0.256 *P*=0.7977; b1 vs b3: *Z* = 0.007 *P*=0.9938; b2 vs b3: *Z* = 0.455 *P*=0.6492; c1 vs c2: *Z* = 2.973 *P*=0.0029; c1 vs c3: *Z* = 0.076 *P*=0.9387; c2 vs c3: *Z* = 3.005 *P*=0.0027; d1vsd2: *Z* = 1.003 *P*=0.3160; d1 vs d3: *Z* = 0.097 *P*=0.92222; d2 vs d3: *Z* = 1.007 *P*=0.3410.

**Table 9:** Implications for optimal marker combinations for lung cancer screening by ROC curves and logistic regression analysis.

Factors	Cutoff	SEN	SPE	AUC	*P*
CEA + CA125	0.447	0.676	0.846	0.821	<0.001
CEA + NSE	0.393	0.645	0.766	0.723	<0.001

**Table 10:** Tumor marker research results.

Healthy		Lung cancer	
Tumor marker	*P*	Tumor marker	*P*
CEA	<0.001	CEA	<0.001
CA125	0.003	CA125	0.46
CY211	0.023	CY211	0.23
GRP	<0.001	GRP	<0.001
NSE	<0.001	NSE	<0.001
CEA + CA125	<0.001	CEA + NSE	<0.001

## Data Availability

Data used to support the findings of this study are available from the corresponding author upon request.

## Additional Points

*Limitations*. Although this study holds significance in detecting cancer at an early stage, aiding in the identification of cancer in asymptomatic individuals, aiding in the identification of cancer in asymptomatic individuals, and facilitating earlier diagnosis, there are several limitations to consider. First, due to the cross-sectional survey design employed in this study, establishing causal relationships between the variables was not possible. Therefore, future research utilizing a longitudinal design will be necessary to establish the causal relationships between these variables. Second, this study was conducted solely in a city in Liaoning province, located in the northeastern region of the country. Consequently, caution should be exercised when generalizing the present results to other regions with different conditions until the findings are replicated in diverse settings. Nevertheless, we did not further classify patients with lung cancer. Several studies have found that serum tumor markers are helpful in the differential diagnosis of pathological lung cancer types [42]. We believe that after diagnosing suspected cancer using serology and imaging, tissue biopsy should be performed to confirm the histological classification of lung cancer based on morphology and special stains [43]. Single serological markers are of little value for predicting the histological subtypes of lung cancer [44]. Finally, biological variability existed among individual patient samples and a wide range of biomarker concentrations across all comparison patients. Inferring diagnostic patterns unique to lung cancer status is a challenge; therefore, standardization of tumor marker analysis must be addressed. Future research will focus on the histopathological classification of lung cancer in patients using tumor markers. This will aid clinicians in treating lung cancer based on changes in tumor marker concentrations.
